# Supplementary material for: Structure of the flotillin complex in a native membrane environment
Source: Proc Natl Acad Sci U S A. 2024 Jul 10;121(29):e2409334121. doi: 10.1073/pnas.2409334121 (PMC11260169; doi:10.1073/pnas.2409334121)
Supplement: Supplementary file 1 — Appendix 01 (PDF) [file pnas.2409334121.sapp.pdf]

**Supporting Information for**

# Structure of the flotillin complex in a native membrane environment

Ziao Fu and Roderick MacKinnon[1]

Laboratory of Molecular Neurobiology and Biophysics, The Rockefeller University, New York, NY, 10065
HHMI, The Rockefeller University, New York, NY, 10065

To whom correspondence may be addressed. Email: mackinn@rockefeller.edu.

**This PDF file includes:**

Supporting text
Figures S1 to S6
Tables S1 to S2

**Fig. S1. Dimension and Shape Comparison between the HlfK/C-FtsH Protein Complex and the Flotillin Complex**

**(A)** Two-dimensional projections were generated from the cryo-electron microscopy map (EMD-32002) of the HlfK/C/FtsH protein complex. The projections were re-centered and lowpass filtered to 20Å. The scale bar represents 20 nm.

**(B-D)** Exemplar cropped micrographs showcase the Flotillin complex observed ouside (B), inside (C), and bound to two (D) native membrane vesicles of HEK293 GnTI$^{-}$ cells. The scale bar represents 20 nm.

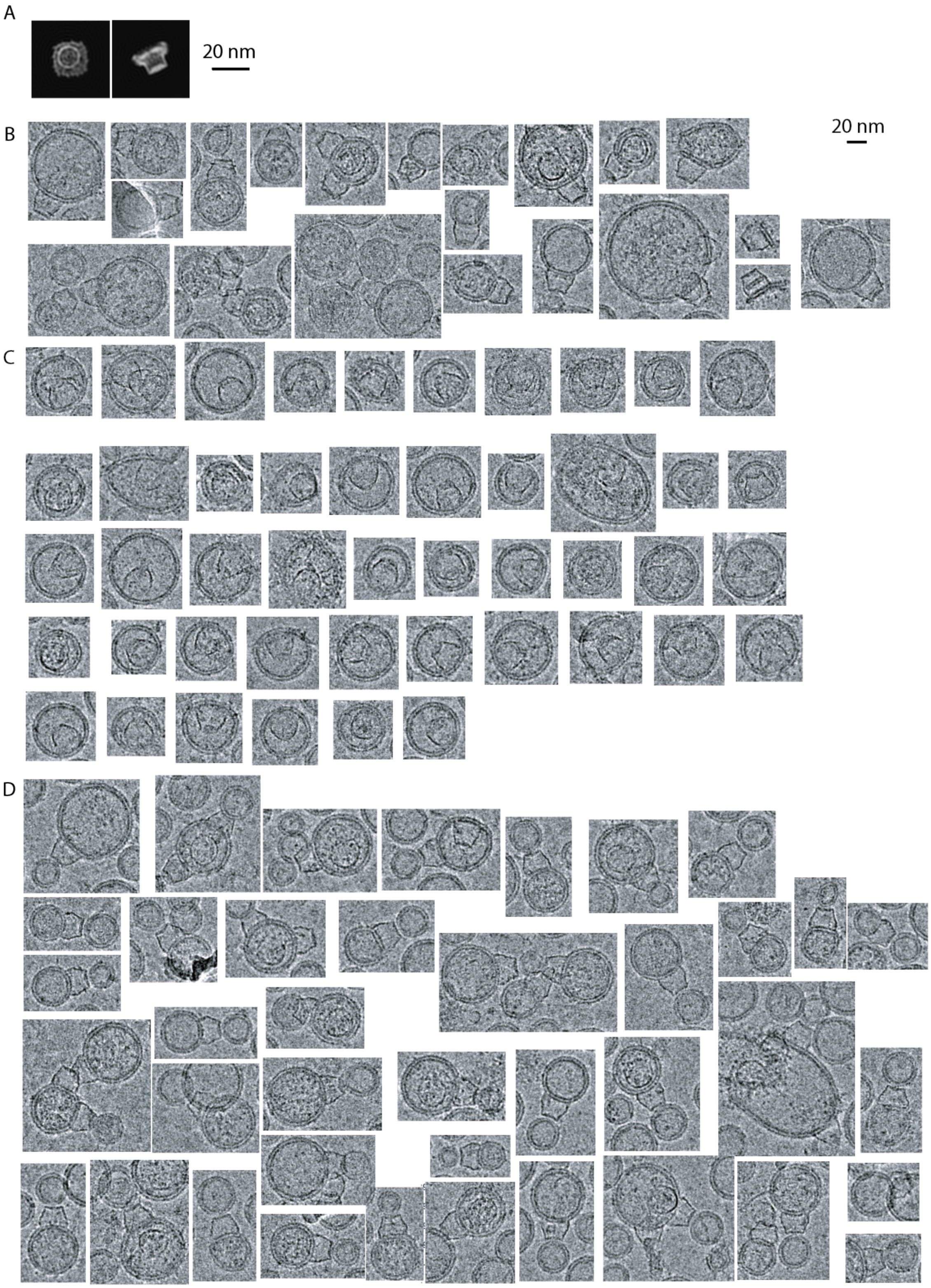
A
20 nm
B
20 nm
C
D

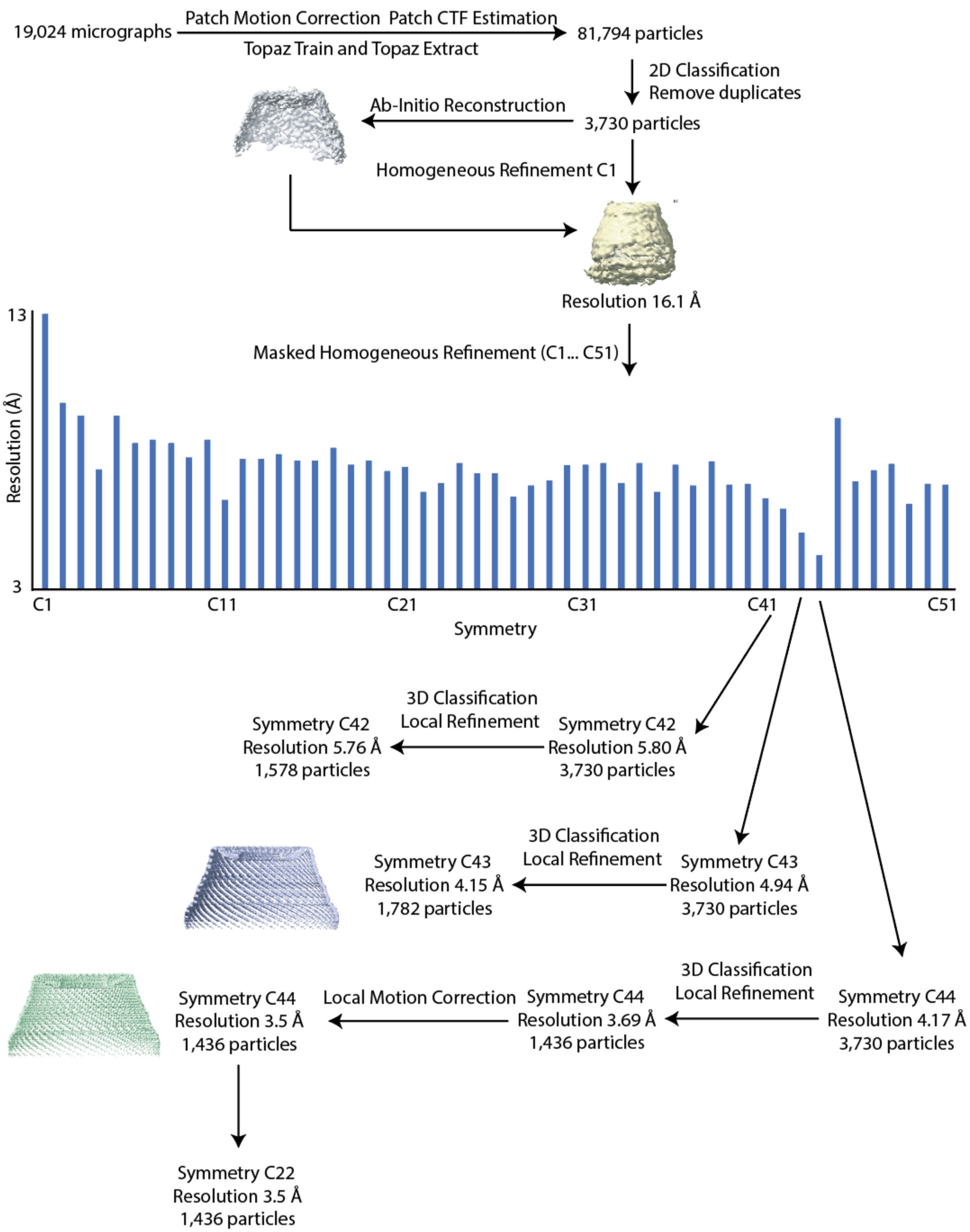


**Fig S2. Cryo-EM data processing procedure**

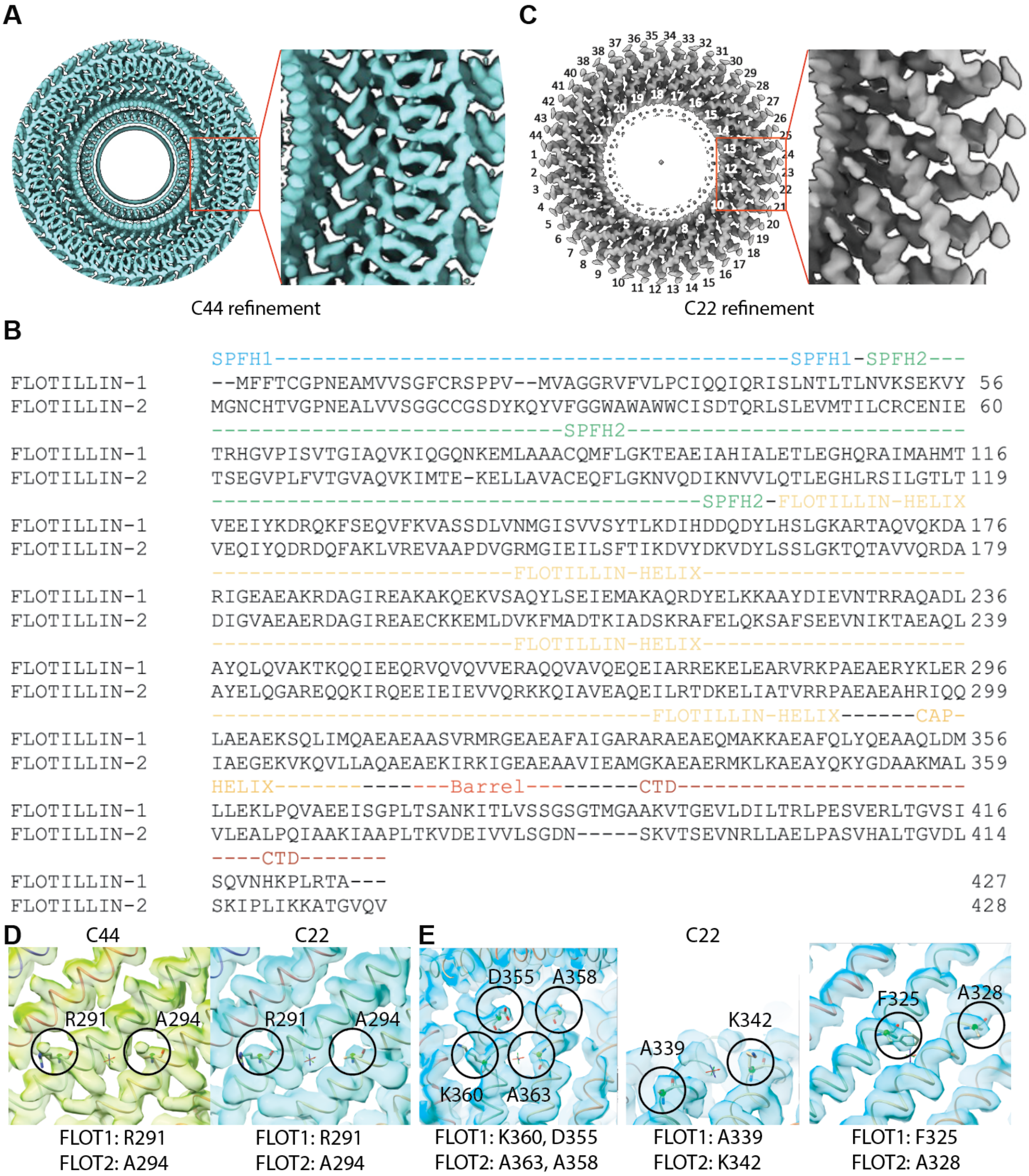
A
C44 refinement
C
C22 refinement
B
SPFH1--------------------------------------SPFH1-SPFH2---
FLOTILLIN-1 --MFFTCGPNEAMVVSGFCRSPPV--MVAGGRVFVLPCIQQIQRISLNTLTLNVKSEKVY 56
FLOTILLIN-2 MGNCHTVGPNEALVVSGGCCGSDYKQYVFGGWAWAWWCISDTQRLSLEVMTILCRCENIE 60
----------------------------SPFH2----------------------------
FLOTILLIN-1 TRHGVPISVTGIAQVKIQGQNKEMLAAACQMFLGKTEAEIAHIALETLEGHQRAIMAHMT 116
FLOTILLIN-2 TSEGVPLFVTGVAQVKIMTE-KELLAVACEQFLGKNVQDIKNVVLQTLEGHLRSILGTLT 119
---------------------------------------SPFH2-FLOTILLIN-HELIX
FLOTILLIN-1 VEEIYKDRQKFSEQVFKVASSDLVNMGISVVSYTLKDIHDDQDYLHSLGKARTAQVQKDA 176
FLOTILLIN-2 VEQIYQDRDQFAKLVREVAAPDVGRMGIEILSFTIKDVYDKVDYLSSLGKTQTAVVQRDA 179
------------------------FLOTILLIN-HELIX---------------------
FLOTILLIN-1 RIGEAEAKRDAGIREAKAKQEKVSAQYLSEIEMAKAQRDYELKKAAYDIEVNTRRAQADL 236
FLOTILLIN-2 DIGVAEAERDAGIREAECKKEMLDVKFMADTKIADSKRAFELQKSAFSEEVNIKTAEAQL 239
------------------------FLOTILLIN-HELIX---------------------
FLOTILLIN-1 AYQLQVAKTKQQIEEQRVQVQVVERAQQVAVQEQEIARREKELEARVRKPAEAERYKLER 296
FLOTILLIN-2 AYELQGAREQQKIRQEEIEIEVVQRKKQIAVEAQEILRTDKELIATVRRPAEAEAHRIQQ 299
-----------------------------------FLOTILLIN-HELIX------CAP-
FLOTILLIN-1 LAEAEKSQLIMQAEAEAASVRMRGEAEAFAIGARARAEAEQMAKKAEAFQLYQEAAQLDM 356
FLOTILLIN-2 IAEGEKVKQVLLAQAEAEKIRKIGEAEAAVIEAMGKAEAERMKLKAEAYQKYGDAAKMAL 359
HELIX-----------Barrel-----------CTD-----------------------
FLOTILLIN-1 LLEKLPQVAEEISGPLTSANKITLVSSGSGTMGAAKVTGEVLDILTRLPESVERLTGVSI 416
FLOTILLIN-2 VLEALPQIAAKIAAPLTKVDEIVVLSGDN-----SKVTSEVNRLLAELPASVHALTGVDL 414
----CTD-------
FLOTILLIN-1 SQVNHKPLRTA--- 427
FLOTILLIN-2 SKIPLIKKATGVQV 428
D
C44
C22
R291
A294
FLOT1: R291
FLOT2: A294
FLOT1: R291
FLOT2: A294
E
C22
D355
A358
K360
A363
K342
A339
F325
A328
FLOT1: K360, D355
FLOT2: A363, A358
FLOT1: A339
FLOT2: K342
FLOT1: F325
FLOT2: A328

**Fig. S3. Symmetry of the Flotillin Complex.**

**(A)** The cryo-EM map of Flotillin's narrow end region, reconstructed with C44 symmetry, exhibits a bottom view, revealing discontinuous density.

**(B)** Sequence alignment of Flotillin-1 and Flotillin-2.

**(C)** Bottom view of the narrow end of the Flotillin's cryo-EM map reconstructed with C22 symmetry, showcasing well-resolved helical density. One layer comprises 44 helices (indicated with 1-44 in black), while the other layer contains 22 helices (indicated with 1-22 in white).

**(D)** In the identical region of the cryo-EM map reconstructed with either C44 or C22 symmetry, the sidechain density becomes apparent, enabling the differentiation of Flotillin-1 and Flotillin-2. Circles emphasize the side chain exhibiting the most significant difference between Flotillin-1and Flotillin-2—specifically, R291 in Flotillin-1and A294 in Flotillin-2 at the corresponding position. The density, which was averaged out and fragmented under C44 symmetry, becomes distinctly resolved when employing C22 symmetry.

**(E)** Representative regions of the cryo-EM map reconstructed with C22 symmetry, demonstrating distinct side chain density on the Flotillin-1and Flotillin-2 subunits.

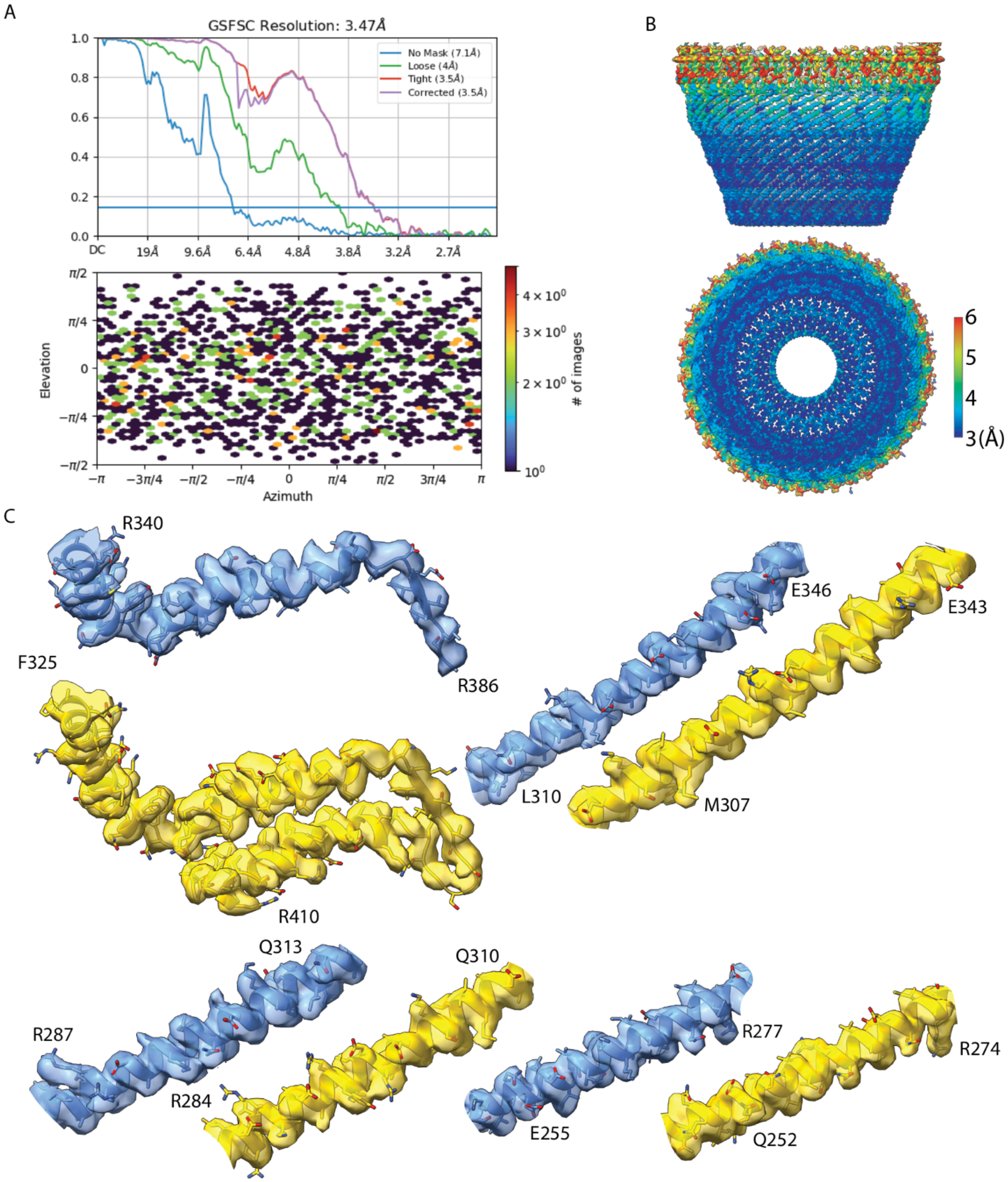


**Fig. S4. Resolution Estimation of the Final Density Map of the Flotillin Complex.** **(A)** Fourier shell correlation (FSC) curves of the final map and orientation distribution plot. At FSC gold-standard 0.143 cutoff, the estimated resolution is 3.5 Å. **(B)** Local resolution estimation of the final cryo-EM map. **(C)** Local densities of regions of Flotillin-1 (yellow) and Flotillin-2 (blue).

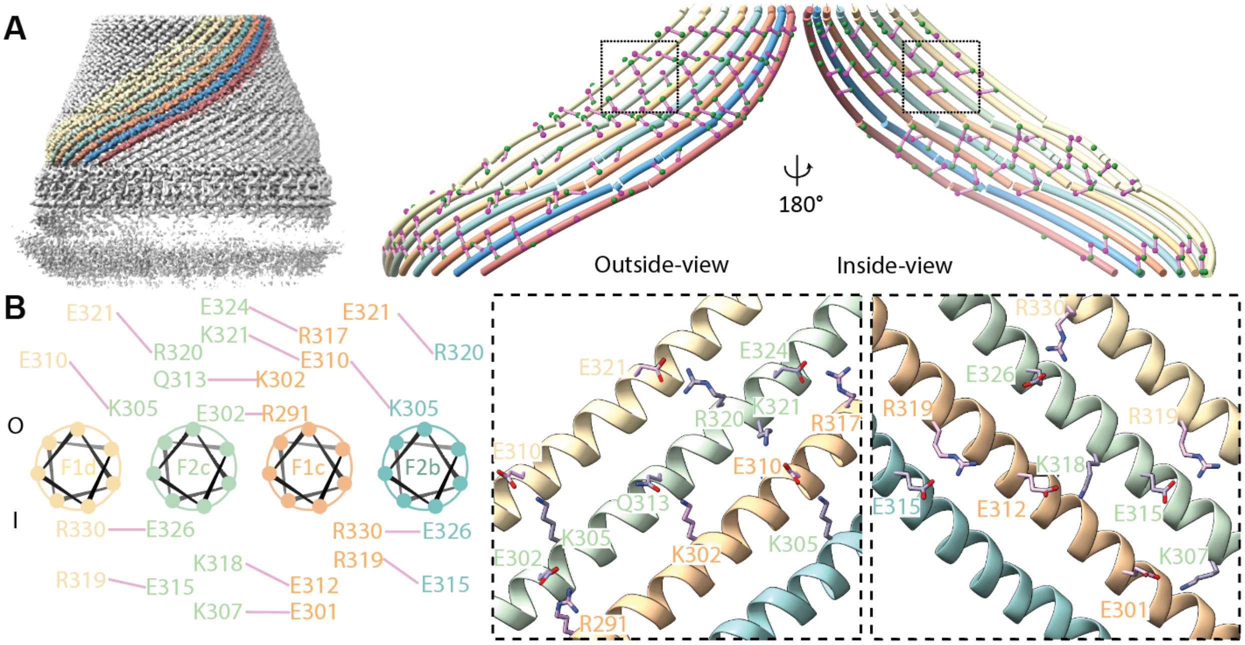


C

```
Flotillin-1                                                  YLHSLGKARTAQVQKDA        176
Flotillin-2                                                  YLSSLGKTQTAVVQRDA        179

Flotillin-1     RIGEAEAKRDAGIREAKAKQEKVSAQYLSEIEMAKAQRDYELKKAAYDIEVNTRRAQADL        236
Flotillin-2     DIGVAEAERDAGIREAECKKEMLDVKFMADTKIADSKRAFELQKSAFSEEVNIKTAEAQL        239

Flotillin-1     AYQLQVAKTKQQIEEQRVQVQVVERAQQVAVQEQEIARREKELEARVRKPAEAERYKLER        296
Flotillin-2     AYELQGAREQQKIRQEEIEIEVVQRKKQIAVEAQEILRTDKELIATVRRPAEAEAHRIQQ        299

Flotillin-1     LAEAEKSQLIMQAEAEAASVRMRGEAEAFAIGARARAEAEQMAKKAEAFQ                  356
Flotillin-2     IAEGEKVKQVLLAQAEAEKIRKIGEAEAAVIEAMGKAEAERMKLKAEAYQ                  359
```

**Fig. S5. Structure and Organization of Wall Regions in the Flotillin Complex.**

**(A)** Side view of the Flotillin complex with eight Wall helices separately colored on the left. On the right, both outside-view and inside-view of the eight Wall helices highlight charged residues involved in forming ionized hydrogen bonds (colored in green and magenta), with interactions represented by solid purple lines connecting the alpha-carbons of interacting amino acids. **(B)** Wheel plot illustrating amino acids from four neighboring subunits involved in inter-subunit ionized hydrogen bonds. The top part shows residues outside the Flotillin complex (labeled as O), and the bottom part shows residues on the inside (labeled as I). Dashed box regions correspond to the zoomed-in view from (A), displaying detailed interactions between charged amino acids involved in ionized hydrogen bonding. **(C)** Sequence alignment of Flotillin's wall helices.

| Uniport entry code | Protein | Organism | Amino acids | Domains |
|---|---|---|---|---|
| A0A6A4LHZ6 | Flotillin-like | Rhododendron williamsianum | 871 | DNA-binding pseudobarrel domain |
| A0A5C6NEC7 | Flotillin-1 | Takifugu flavidus (sansaifugu) | 2335 | Kinesin motor domain |
| L9KYH7 | Mediator of DNA damage checkpoint protein 1 | Tupaia chinensis (Chinese tree shrew) | 2327 | BRCT domain |
| A0A094CTU6 | RING-type domain-containing protein | Pseudogymnoascus sp. VKM F-4516 (FW-969) | 2146 | RING-H2_Vps11； MFS_Mch1p_like |
| T0M7H4 | Uncharacterized protein | Camelus ferus (Wild bactrian camel) (Camelus bactrianus ferus) | 2139 | |
| A0A6A5DRU9 | Putative ank repeat-containing | Schistosoma haematobium (Blood fluke) | 1857 | Ankyrin repeat-containing domain;P-loop containing nucleotide triphosphate hydrolases |
| A0A1I8GQF9 | Flotillin-1 | Macrostomum lignano | 1538 | Nucleic acid-binding proteins |
| A0A1I8II45 | Flotillin-1 | Macrostomum lignano | 1175 | mostly disordered loop |
| A0A1I8ILN5 | PHB domain-containing protein | Macrostomum lignano | 1175 | unknown function |
| A0A1I8FK26 | PHB domain-containing protein | Macrostomum lignano | 735 | Zn peptidases |
| A0A1I8ITQ5 | PHB domain-containing protein | Macrostomum lignano | 638 | MFS transporter superfamily |
| A0A1I8GKQ6 | Flotillin-1 | Macrostomum lignano | 539 | SPFH2 domain |
| E2BW52 | Flotillin-1 | Harpegnathos saltator (Jerdon's jumping ant) | 1191 | S-adenosyl-L-methionine-dependent methyltransferase superfamily |
| A0A3M0KUG2 | Era-type G domain-containing protein | Hirundo rustica rustica | 1220 | N:retinol dehydrogenase C:Era-type guanine nucleotide-binding (G) domain profile C:type II K-homology (KH) RNA-binding domain |
| A0A182JDQ7 | Sulfhydryl oxidase | Anopheles atroparvus (European mosquito) | 1388 | ERV/ALR sulfhydryl oxidase;Steroid 5-alpha reductase C-terminal |
| A0A7G2FMU5 | 4-alpha-glucanotransferase | Arabidopsis thaliana (Mouse-ear cress) | 1048 | Glycoside_hydrolase |
| A0A517LQQ4 | DDHD domain-containing protein | Venturia effusa | 1508 | PHOSPHOLIPASE DDHD1 |
| A0A835G3R8 | Band 7 domain-containing protein | Spodoptera exigua (Beet armyworm) (Noctua fulgens) | 1484 | DUF916 |
| M7CAV2 | Flotillin-2 | Chelonia mydas (Green sea-turtle) (Chelonia agassizi) | 1440 | forkhead associated (FHA) domain found in PHD finger protein 12 (PHF12) and similar proteins;short chain dehydrogenase; |
| A0A6A2YXC7 | Flotillin-like protein 1 | Hibiscus syriacus (Rose of Sharon) | 1334 | Ribonuclease H-like superfamily/Ribonuclease H |
| A0A498L6I4 | polypeptide N-acetylgalactosaminyltransferase | Labeo rohita (Indian major carp) (Cyprinus rohita) | 1203 | polypeptide N-acetylgalactosaminyltransferase |
| A0A8X8ZUG4 | Ubiquitin-like domain-containing protein | Salvia splendens (Scarlet sage) | 1197 | Ubiquitin-like |
| A0A3L6ELD6 | Flotillin-like protein 1 | Zea mays (Maize) | 1118 | The CRM domain: An RNA binding module derived from an ancient ribosome-associated protein |
| A0A812WY35 | YqiK protein | Symbiodinium pilosum (Dinoflagellate) | 1108 | PspA_IM30 |
| A0A812RIR5 | Adenylosuccinate synthetase | Symbiodinium pilosum (Dinoflagellate) | 2470 | Histidyl-tRNA synthetase； Adenylosuccinate synthetase； 3-Oxoacyl-[acyl-carrier-protein (ACP)] synthase III； |
| A0A8S2B2X3 | 4-alpha-glucanotransferase | Arabidopsis arenosa (Sand rock-cress) (Cardaminopsis arenosa) | 1099 | 4-alpha-glucanotransferase |
| A0A7R8H3C7 | flotillin-1 | Lepeophtheirus salmonis (Salmon louse) | 882 | Cytidine Deaminase, domain 2; Restriction enzyme adenine methylase associated |

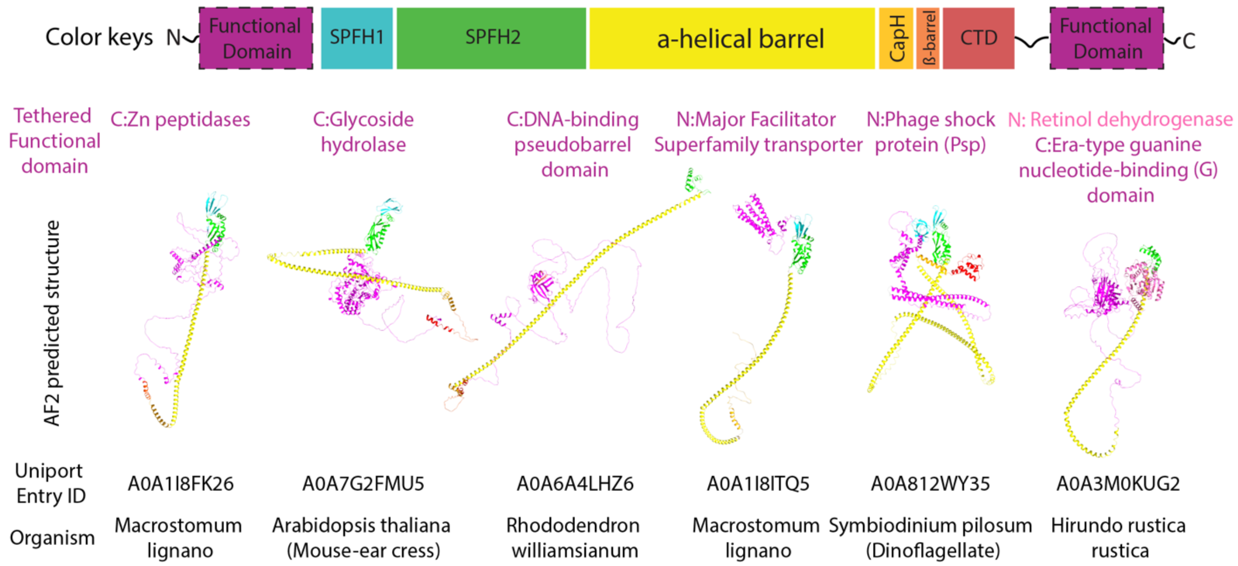


**Fig. S6. Tethered Functional Domains in Flotillin-Like Proteins**

Proteins with conserved Flotillin-like structures and functional domains tethered to their N- or C-termini, as identified in the UniProt database. Each entry includes the UniProt code, protein name, organism, total amino acids, and domain names. The figure illustrates representative Flotillin-like proteins with tethered functional domains; these are colored based on their location at the N- or C-terminus (shown in pink) and labeled accordingly. Below each predicted AlphaFold2 structure, the corresponding UniProt entry ID and organism name are displayed.

## Table S1. Western Blot Analysis of Stomatin, Stomatin-like Protein 2, Flotillin-1 and Flotillin-2 in HEK293 GnTI⁻ Cells

| Protein name | Stomatin | Stomatin-like protein 2 | Flotillin-1 | Flotillin-2 |
|---|---|---|---|---|
| Molecular Weight (kDa) | 31.7 | 38.5 | 47.7 | 47.1 |
| Western Blot | 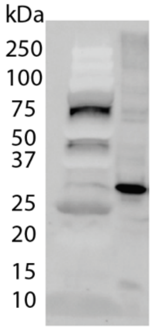  | 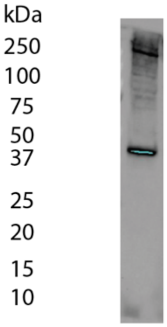  | 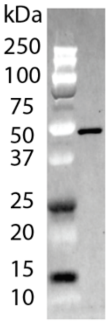  | 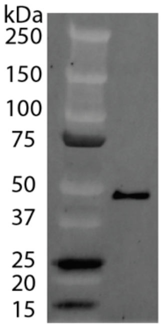  |

**Table S2. Cryo-EM Data Collection, Refinement and Validation Statistics**

| | Flotillin complex |
|---|---|
| **Data collection and processing** | |
| Magnification | 105kx |
| Voltage (kV) | 300 |
| Electron exposure (e-/Å2) | 42 |
| Defocus range (μm) | 1.5-2 |
| Pixel size (Å) | 1.196 |
| Symmetry imposed | C22 |
| Final particle images (no.) | 1,436 |
| Map resolution (Å) | 3.5 |
| FSC threshold | 0.143 |
| | |
| **Refinement** | |
| Map sharpening B factor (Å2) | -100 |
| | |
| **Model composition** | |
| Chains | 12 |
| Atoms | 72978 (Hydrogens: 36258) |
| Residues | Protein: 4944 Nucleotide: 0 |
| Water | 0 |
| Ligands | 0 |
| | |
| Bond lengths (Å) | 0.011 |
| Bond angles (°) | 2.073 |
| | |
| **Validation** | |
| MolProbity score | 1.36 |
| Clashscore | 2.06 |
| Rotamers outliers (%) | 1.45 |
| | |
| **Ramachandran plot** | |
| Favored (%) | 96.10 |
| Allowed (%) | 3.29 |
| Outliers (%) | 0.61 |
